# Environmental Factors Associated with Severe Motorcycle Crash Injury in University Neighborhoods: A Multicenter Study in Taiwan

**DOI:** 10.3390/ijerph191610274

**Published:** 2022-08-18

**Authors:** Heng-Yu Lin, Jian-Sing Li, Chih-Wei Pai, Wu-Chien Chien, Wen-Cheng Huang, Chin-Wang Hsu, Chia-Chieh Wu, Shih-Hsiang Yu, Wen-Ta Chiu, Carlos Lam

**Affiliations:** 1 School of Medicine, College of Medicine, Taipei Medical University, Taipei 11031, Taiwan; 2 Department of Emergency Medicine, Taipei Medical University Hospital, Taipei 11031, Taiwan; 3 Graduate Institute of Injury Prevention and Control, College of Public Health, Taipei Medical University, Taipei 11031, Taiwan; 4 Department of Medical Research, Tri-Service General Hospital, National Defense Medical Center, Taipei 11490, Taiwan; 5 Graduate Institute of Life Sciences, National Defense Medical Center, Taipei 11490, Taiwan; 6 School of Public Health, National Defense Medical Center, Taipei 11490, Taiwan; 7 Taiwanese Injury Prevention and Safety Promotion Association, Taipei 11490, Taiwan; 8 Emergency Department, Department of Emergency and Critical Care Medicine, Wan Fang Hospital, Taipei Medical University, Taipei 11696, Taiwan; 9 Department of Emergency, School of Medicine, College of Medicine, Taipei Medical University, Taipei 11031, Taiwan; 10 Center for Education in Medical Simulation, Taipei Medical University, Taipei 11031, Taiwan; 11 Department of Education and Humanities in Medicine, School of Medicine, College of Medicine, Taipei Medical University, Taipei 11031, Taiwan; 12 Emergency Department, Wan Fang Hospital, Taipei Medical University, Taipei 11696, Taiwan; 13 Institute of Transportation, Ministry of Transportation and Communications, Taipei 10548, Taiwan; 14 AHMC Health System, Alhambra, CA 91801, USA

**Keywords:** university neighborhood, environmental factor, motorcyclist, injury severity, young adult

## Abstract

University neighborhoods in Taiwan have high-volume traffic, which may increase motorcyclists’ risk of injury. However, few studies have analyzed the environmental factors affecting motorcycle crash injury severity in university neighborhoods. In this multicenter cross-sectional study, we explored the factors that increase the severity of such injuries, especially among young adults. We retrospectively connected hospital data to the Police Traffic Accident Dataset. Areas within 500 m of a university were considered university neighborhoods. We analyzed 4751 patients, including 513 with severe injury (injury severity score ≥ 8). Multivariate analysis revealed that female sex, age ≥ 45 years, drunk driving, early morning driving, flashing signals, and single-motorcycle crashes were risk factors for severe injury. Among patients aged 18–24 years, female sex, late-night and afternoon driving, and flashing signals were risk factors. Adverse weather did not increase the risk. Time to hospital was a protective factor, reflecting the effectiveness of urban emergency medical services. Lifestyle habits among young adults, such as drunk driving incidents and afternoon and late-night driving, were also explored. We discovered that understanding chaotic traffic in the early morning, flashing signals at the intersections, and roadside obstacles is key for mitigating injury severity from motorcycle crashes in university neighborhoods.

## 1. Introduction

Road traffic injuries (RTIs) are the most common cause of death and injury among young adults worldwide [1]. In developed countries, young motorcyclists have a high mortality and morbidity rate, resulting in considerable functional impairment among 23.4% of injured patients [2,3]. In the United States, university students who ride a motorcycle have high injury rates [4]. In developed countries, motorcycles are commonly used for leisure. Nevertheless, in Taiwan and numerous developing countries, light motorcycles with cylinder capacities typically less than 150 cc. are used for commuting and daily transportation [5]. Because of the convenience and affordability, 66% of Taiwanese university students use light motorcycles for their daily transportation [6].

Fatalities due to motorcycle crashes are particularly common among university students [7]. The Ministry of Education statistical report showed that a total of 1665 university students were killed in road traffic accidents (RTAs) between 2009 and 2018, and more than 90% of fatalities were caused by motorcycle crashes [7,8]. Almost 93% of the RTIs among Taiwanese university students were due to motorcycle crashes [9]. However, few studies have identified the associated risk factors. Studies from Taiwan and Thailand have reported that a lack of experience, drunk driving, and violations of traffic rules were significant risk factors for RTIs among university students [10,11]. University students in Thailand considered elements in the environments surrounding campuses to be considerable risk factors for RTAs. Dangerous elements such as lack of regulatory signs and street lighting, water retained on roads, and bumpy roads contribute to hazardous driving conditions in university neighborhoods [11]. The risk of RTIs is also increased on rural roads, during inclement weather, and in areas with a high number of schools [12,13].

**Figure 1:**
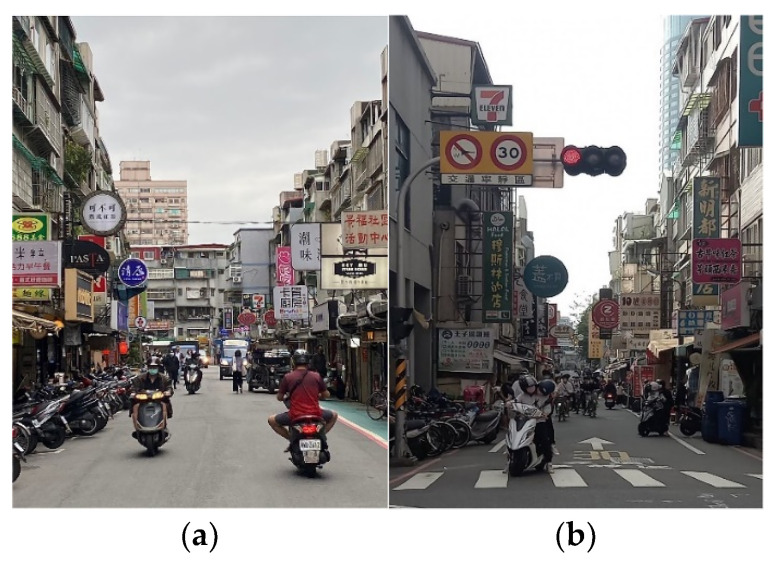
University neighborhoods in Taiwan. (**a**) A university neighborhood in Taipei City. (**b**) A university neighborhood in Tainan City. Source: Pictures taken by the author (H.-Y.L.).

Most studies on RTIs in school neighborhoods have focused on primary and secondary schools and reported school neighborhoods as a risk factor for serious and fatal RTIs [14,15]. However, few studies have analyzed motorcycle crashes in university neighborhoods. In Taiwan, university neighborhoods often have numerous restaurants and small stores, which are the main locations for students to shop and dine. These highly commercialized areas also attract residents; they resemble commercial districts more than school districts (Figure 1) [16]. The high volume of road users may increase the severity of RTIs [17].

Therefore, by using a dataset established for a road safety study sponsored by the Ministry of Transportation and Communications (MOTC) [18], we carried out a multicenter cross-sectional study to evaluate the risk factors for severe RTIs among motorcyclists in university neighborhoods. The dataset mentioned above for analysis does not contain information on university student status. However, the proportion of Taiwanese high school graduates attending universities was almost 90% in 2017 [19]. The official statistics from the MOTC also reported that most of the casualties in hotspots around universities were young adults aged 20–24 years [20]. Thus, we extracted the data of patients aged 18–24 years as a surrogate for university students and performed a subgroup analysis to evaluate the risk factors for severe motorcycle crash injury among young adults. Our results can help authorities mitigate the risk of motorcycle crash injuries in university neighborhoods, especially among young adults.

## 2. Materials and Methods

### 2.1. Data Source

**Figure 2:**
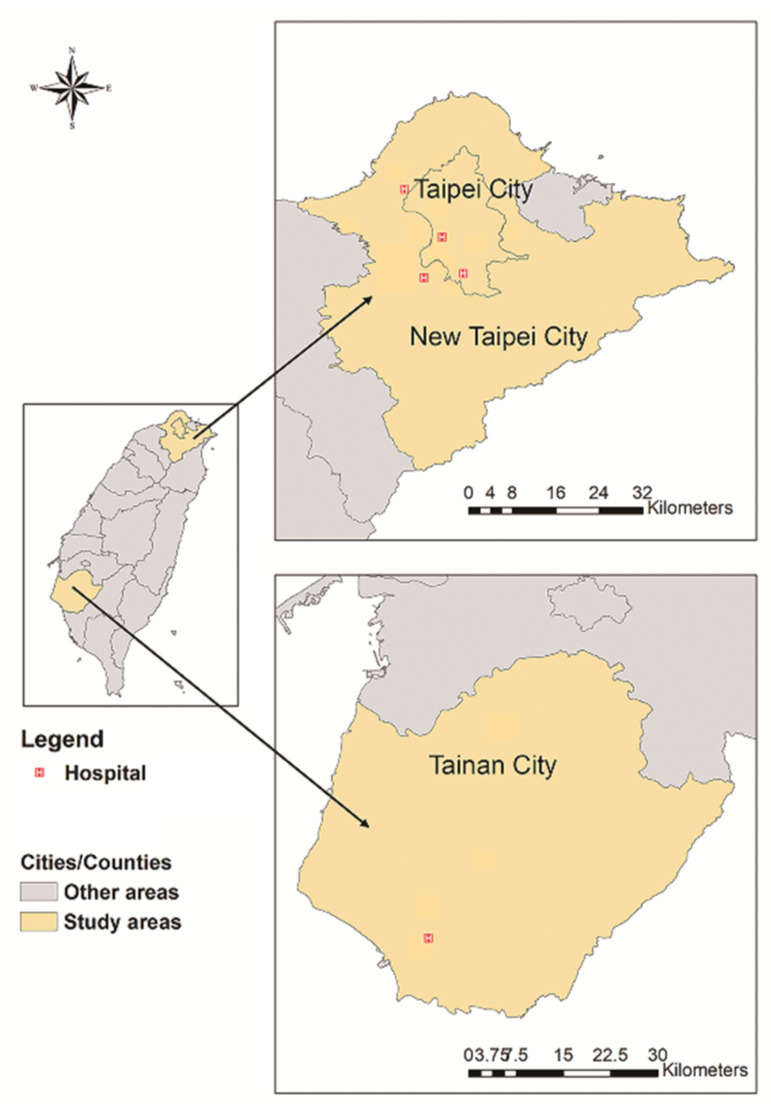
Locations of participating hospitals.

The hospitals enrolled in our study included the following: Shuang Ho Hospital and Mackay Memorial Hospital (Tamsui Branch) in the southwest and northeast regions of New Taipei City, with a population of 401,086 and 184,660, respectively; Wan Fang Hospital and Mackay Memorial Hospital (Taipei Branch) in the southern and central regions of Taipei City, with a population of 256,942 and 210,447, respectively; National Cheng Kung University Hospital in the northern region of Tainan City, with a population of 126,579. All hospitals (4600 beds in total) are located in urban regions and accredited as advanced emergency responsibility hospitals in Taiwan, delivering extensive treatment for injured patients (Figure 2). We retrospectively reviewed the hospital data of the patients treated in the emergency departments (EDs) for RTIs between 1 January and 31 December 2017.

The hospital data were linked to the Police Traffic Accident Dataset (PTAD) by using national identification numbers to identify patients treated in the ED for RTIs. The identification numbers were deleted after this connection was established. The data in the PTAD are recorded by the police at the location of an RTA and include information such as time and location, road and weather conditions, type of crash, cause, and whether drunk driving was involved. Information regarding persons involved includes demographic data, type of road user (auto occupants, motorcyclists, bicyclists, or pedestrians), and licentiateship (driving with or without a legal license). To ensure the causal relationship between the RTAs and the ED visits, patients who presented to the ED more than 24 h after the RTA were excluded. Only the first visit records were retrieved for patients who had multiple ED visits after the RTI. The institutional review board of each participating hospital approved this study (16MMHIS168e, N201510012, and A-ER-105-401): 16MMHIS168e for Mackay Memorial Hospital (Taipei and Tamsui Branch); N201510012 for Taipei Medical University (Shuang Ho Hospital and Wan Fang Hospital); A-ER-105-401 for National Cheng Kung University Hospital. Because of the high number of patients and the retrospective and noninterventional nature of the study, informed consent was not sought. This study was performed in accordance with the Strengthening the Reporting of Observational Studies in Epidemiology guidelines.

### 2.2. Study Design and Participants

The risk and patterns of injuries between drivers and pillion passengers involved in motorcycle crashes might be different [21]. Previous research pointed out that the seating position among motorcycle passengers was associated with their injury pattern or severity [22]. To avoid bias due to contamination and improve the homogeneity of the analysis data, we selected only the victims who were driving. Geographic information system platforms, including Google Map application programming interfaces [23] and Taiwan Geospatial One Stop [24], were used to geocode the RTA locations in the PTAD. Jeng et al. investigated the movement area of university students [25] in Taiwan through behavior mapping and activity path analysis and revealed that the area with the most movement was within 500 m of universities, accounting for more than 80% of movement [26,27]. Therefore, a 500-m radius outside the universities’ main gates was defined as the university neighborhood. Motorcycle crashes within university campuses are not recorded in the PTAD. This study only used universities in areas covered by emergency medical services, which are responsible for transporting patients to the five participating hospitals’ ED. The RTA patterns in rural areas differ from those in urban areas [12,28]. In Taiwan, urbanization is categorized into seven levels [29], with levels 4–7 indicating rural areas [30]. Therefore, all RTAs in rural areas (levels 4–7) were excluded to reduce heterogeneity. We also excluded RTAs occurring around military colleges and police academies because the daily movement of their students differs from those of civilian students.

### 2.3. Measurements

The injury severity score (ISS) is generally used by medical professionals to evaluate injury severity. This study used patients’ ISS as an outcome measure and retrieved it from the hospital data. The ISS has calculated the sum of the squares of the three highest abbreviated injury scale (AIS) scores [31]. The AIS divides the human anatomical region into six portions: the head and neck, face, thorax, abdomen, extremities, and external [32]. The AIS scores are from 1 to 6, with higher scores indicating more serious injuries. Advanced emergency responsibility hospitals in Taiwan, equivalent to trauma centers in the United States, must record the ISS among trauma patients discharged from the hospital. However, this is not applicable for patients discharged from the ED without hospitalization. For these patients, ICDPIC-R, a validated software, was applied to convert their *International Classification of Diseases, 10th Revision, Clinical Modification* codes in the ED to ISS [33].

The patients’ age, which was divided into four groups (≤24, 25–44, 45–64, and ≥65 years), and sex were retrieved from the hospital data. Independent variables retrieved from the PTAD were drunk driving, cylinder capacities, license status, weather, day of week, time of crash, rush hour, speed limit, road alignment, road surface, sight distance, signal status, and collision partner.

Drunk driving was assumed if alcohol was detected using a breath-testing device or blood test. Cylinder capacities were categorized as <250 cc (light motorcycle) and ≥250 cc (heavy motorcycle). The riders’ license status was categorized as “unlicensed” for those without a valid license and “licensed” for those with a valid license. Weather conditions were categorized as fine or adverse. The day of the week was divided into weekday (Monday to Friday) and weekend (Saturday and Sunday). The time of crash was divided into six groups: late night (12:00 a.m.–03:59 a.m.), early morning (04:00 a.m.–07:59 a.m.), morning (08:00 a.m.–12:00 p.m.), afternoon (12:00 p.m.–3:59 p.m.), evening (4:00 p.m.–7:59 p.m.), and night (8:00 p.m.–11:59 p.m.). The periods from 07:00 a.m. to 09:59 a.m. and from 6:00 p.m. to 8:59 p.m. were considered rush hours. The road conditions were defined as follows: speed limit (≤50 and >50 km/h); road alignment (straight road, curved road, and crossroad/roundabout); road surface (dry and wet/slippery); sight distance (good and bad); and signal status (normal, flashing, and no signal). The road width (meter) was computed using a commercial digital map, ArcGIS 10.2 (Environmental Systems Research Institute, Redlands, CA, USA) and was used to as a surrogate for the local traffic volume [34]. Collision partners comprised three categories: pedestrians, vehicles, and none. The time to hospital (minute) was the time interval between the RTA occurrence and ED arrival, which were respectively retrieved from the PTAD and hospital data.

### 2.4. Statistical Analysis

In this study, we used the dichotomy to approach ISS to achieve injury sensitivity but also because there are statistical problems when ISS is used as a continuous variable. Since the ISS of this study was not a normal distribution (skewness = 3.68, kurtosis = 17.38, *p* < 0.01) and could not be transformed to normal distributions by methods such as log, square root, and inverse logit transformations, applying the mean ISS for statistics might cause misinterpretation [35,36,37].

Most RTIs in Taiwan are not life-threatening injuries [18], so morbidity, rather than mortality, was considered an outcome of interest. Palmer et al. suggested that an ISS cutoff of 8 can be used for patients with more severe injury necessitating hospitalization or intensive care [35]. Thus, ISS ≥ 8 was used to indicate severe injury in this study [38].

We first compared the distribution of injury severities (ISS < 8 or ISS ≥ 8) in each independent variable. Because the dependent variable (outcome measure) was binary (ISS < 8 and ISS ≥ 8), a simple logistic regression was conducted to estimate the association between the independent variables and the outcome measure. The multiple logistic regression analysis was conducted by using the independent variables with *p* < 0.2 in univariate analysis [39,40]. In Taiwan, the proportion of Taiwanese high school graduates attending universities was almost 90% in 2017 [19]. We selected the patients in this age group to achieve a subgroup analysis.

We used odds ratios (ORs) and 95% confidence intervals (CIs) to calculate the risk of severe motorcycle crash injuries in university neighborhoods. A variance inflation factors (VIF) was used to evaluate the collinearity among all covariates in the multiple regression model. Variables with VIF < 5 were selected. The statistical analysis was limited to samples without missing values for a particular variable. A two-sided *p* < 0.05 was considered statistically significant. This statistical analysis was executed using R studio software (Version 1.4.1717; R Studio Inc., Boston, MA, USA).

When conducting the multiple logistic regression analysis, the reference group for each independent variable was selected based on the logic of the normative group or the largest sample size [41]. For example, for the variable of drunk driving, the notable contrast is to determine whether the alcohol-impaired drivers differed in injury severity from the normative group, which also consisted of a larger number of drivers. Thus, the normative group was assigned as a reference group for an easier explanation of their results. Taking the age group as another example, although most casualties in hotspots around universities were young adults [20], those aged 18–24 were the focus of our study. Based on prior findings, middle-aged and older adults were inclined to suffer severe injuries in collisions [5,42]. Therefore, those aged 25–44 were the reference group.

## 3. Results

**Figure 3:**
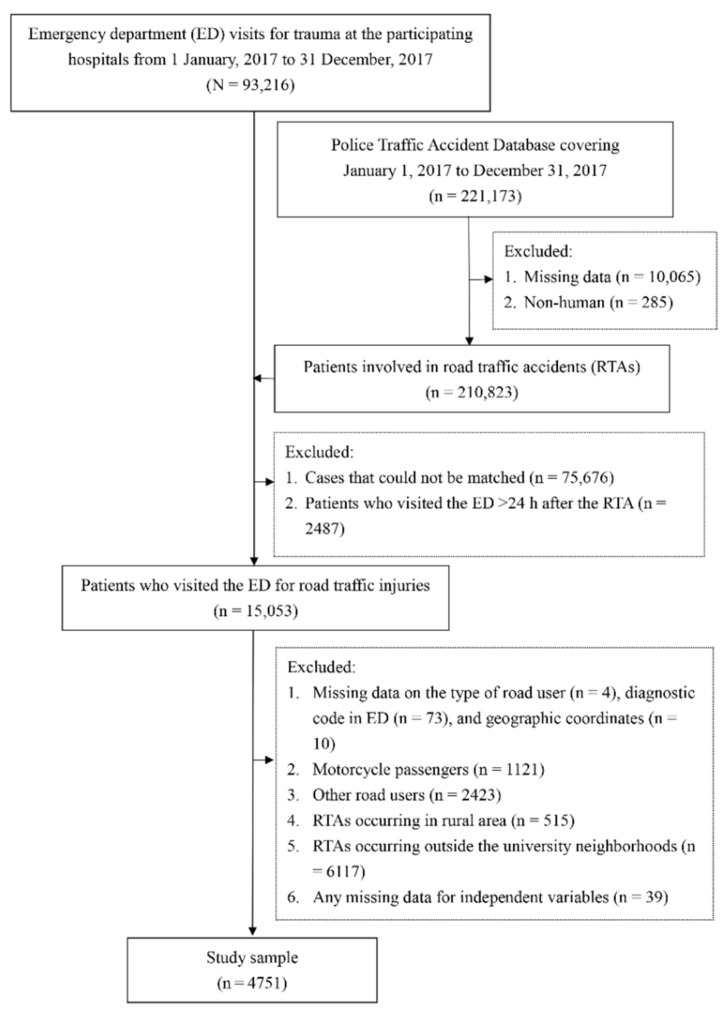
Flow chart for sample selection. ED, emergency department; RTA, road traffic accident.

We enrolled 4751 patients in the study (Figure 3). The mean age was 34.84 years, with 1838 patients (38.69%) being aged 18–24 and 2917 (61.40%) being male. Among these patients, 513 (10.79%) sustained a severe injury (ISS of ≥8).

Table 1 presents the distribution of each independent variable between the two groups: ISS < 8 and ISS ≥ 8. Women (12.54%) had a higher percentage of severe injuries (ISS ≥ 8) than men (9.7%). The percentage of severe injuries in the age group ≥ 65 years (19.53%) was the highest among all age groups. The severe injury percentage of drunk driving (28.57%) was significantly higher than that of sober driving (10.67%). The severe injury percentage of unlicensed driving (15.75%) was also significantly higher than that of licensed driving (10.52%). Regarding weather conditions, the percentage of severe injuries that occurred in adverse weather (9.11%) was significantly lower than that in fine weather (11.47%). In signal status, flashing signal (20.11%) had the highest percentage of severe injuries. Among the collision partners, the highest percentage of severe injuries was single-motorcycle crashes (16.04%), followed by collisions with vehicles (10.52%), and the lowest was collisions with pedestrians (8.67%). Compared with patients with ISS < 8 (33 min), patients with ISS ≥ 8 (28 min) had a shorter time to the hospital.

Based on the results of simple logistic regression, we observed that women were more likely to have an ISS of ≥ 8 than men (OR: 1.335, 95% CI: 1.110–1.605). Among the age group, drivers aged 45–64 and ≥65 years were more likely to sustain a severe injury (OR: 1.605, 95% CI: 1.306–1.972 and OR: 2.114, 95% CI: 1.529–2.921, respectively), whereas those aged < 24 and 25–44 were less likely to obtain a severe injury (OR: 0.756, 95% CI: 0.623–0.917 and OR: 0.723, 95% CI: 0.590–0.886, respectively). Motorcyclists were more likely to obtain a severe injury when they were drunk (OR: 3.352, 95% CI: 1.601–7.019), unlicensed (OR: 1.590, 95% CI: 1.120–2.258), driving between 4:00 a.m. and 7:59 a.m. (OR: 1.379, 95% CI: 1.034–1.838), and involving in single-motorcycle crashes (OR: 1.637, 95% CI: 1.181–2.268). Adverse weather (OR: 0.773, 95% CI: 0.625–0.957), no signal (OR: 0.831, 95% CI: 0.692–0.998), and time to the hospital (OR: 0.984, 95% CI: 0.972–0.995) were negatively associated with severe motorcycle crash injuries in university neighborhoods.

Table 2 presents the results of a multivariate analysis between independent variables and severe injury (ISS ≥ 8). Women had a higher risk of severe injury (OR: 1.315, 95% CI: 1.087–1.592) than did men. The risk of having an ISS of ≥8 was significantly higher among patients aged 45–64 (OR: 1.751, 95% CI: 1.365–2.247) and ≥65 years (OR: 2.547, 95% CI: 1.776–3.653) than among those aged 25–44 years. Drunk driving was a risk factor for severe injury (OR: 2.918, 95% CI: 1.355–6.281). Compared with morning crashes (8:00 a.m.–11:59 a.m.), an early morning crash (4:00 a.m.–7:59 a.m.) was more likely to have an ISS of ≥ 8 (OR: 1.408, 95% CI: 1.010–1.963). Flashing signals were a significant risk factor compared with normal signals (OR: 1.968, 95% CI: 1.324–2.927). Compared with collisions with pedestrians, single-motorcycle crashes (OR: 2.150, 95% CI: 1.112–4.155) were a significant risk factor for an ISS of ≥8. Adverse weather and time to hospital were protective factors (OR: 0.779, 95% CI: 0.626–0.969 and OR: 0.987, 95% CI: 0.976–0.998, respectively).

### Subgroup Analysis of 18–24-Year-Old Patients

Table 3 presents the results of a subgroup analysis between independent variables and severe injury (ISS ≥ 8) among young adults. Women had a significantly higher risk of severe injuries (OR: 1.555, 95% CI: 1.121–2.157). Compared with morning crashes (8:00 a.m.–11:59 a.m.), late night (12:00 a.m.–3:59 a.m.) and afternoon (12:00 p.m.–3:59 p.m.) crashes were risk factors for severe injuries (OR: 2.116, 95% CI: 1.096–4.083 and OR: 1.749, 95% CI: 1.085–2.819, respectively). Compared with normal signals, flashing signals (OR: 2.913, 95% CI: 1.628–5.213) significantly increased severe injury risk. Time to hospital had a protective effect (OR: 0.976, 95% CI: 0.954–0.999).

## 4. Discussion

Some methodological innovation and improvement is worth mentioning. As far as we can tell, no sizable epidemiological studies have been conducted to elucidate the associations of environmental conditions with the severity of motorcycle crashes in university neighborhoods. Questionnaire-based research is commonly used to analyze motorcyclists’ behavior among university students and young drivers [11,12,43], while our study aimed to evaluate the association of environmental conditions with the injury severity caused by motorcycle crashes near the university. No study has clearly defined “university neighborhoods” for road safety; therefore, we considered areas with the most movement of university students, cited by several Taiwanese reports [26,27]. The coordinates obtained after converting the RTA addresses in PTAD by using the geographic information system platforms were essential to identifying motorcycle crashes in university neighborhoods. The patients’ injury data were extracted from the electronic medical records of participating hospitals, ensuring accurate assessment of injury severity, unlike other studies relying on the police-reported crash data [44,45]. Concerning the improvement in methodology, our study only selected motorcycle drivers, and the motorcycle crash injury occurred in urban areas. Furthermore, considering that Taiwan has a high rate of high school graduates attending universities [19], the 18–24-year-old adults injured in motorcycle accidents in university neighborhoods were considered the surrogate of the university students.

Our data indicate that driving motorcycles between 4:00 a.m. and 7:59 a.m. elevated the risk of severe injury, which is consistent with studies in Singapore and Malaysia [46,47]. Besides drunk driving and speeding [46,48], further risk factors for severe injury from motorcycle crashes are also present in the early morning. During this period, university students and commuters living nearby drive through university neighborhoods to class or work, and parents often bring their children to school on motorcycles [49], resulting in a high motorcyclist volume in university neighborhoods. The small breakfast shops popular in Taiwan and the stores preparing for business also create traffic chaos because the vendors and other vehicles often park illegally.

For young adults, driving late at night (12:00 a.m.–3:59 a.m.) and in the afternoon (12:00 p.m.–3:59 p.m.) was associated with a higher risk of severe injury. Late-night driving is common among young drivers, manifesting the different lifestyle habits among the young adults. Thompson et al. suggested that afternoon driving is generally recreational and carries a higher risk of RTIs [50]. Young adults, especially university students, have the luxury of driving for recreation during this period.

Our results indicate that flashing traffic signals in university neighborhoods were significantly associated with severe RTIs. In Taiwan, major roadways usually have normal traffic signals, whereas minor roadways often have flashing traffic signals. These flashing traffic signals are commonly used at intersections with low traffic flow and late at night or early in the morning. A Korean study reported that fatalities and severe injuries increased by 77% when signals were flashing at night [51]. In North Carolina, changing flashing signals at night resulted in a 60% reduction in crashes with injury [52]. We observed more severe injuries late at night and in the early morning, which is when flashing signals are used. The frequency of angle collisions also increased among RTIs in intersections with flashing signals [53]; this causes more severe injuries than does other types of collisions [54]. Most streets in university neighborhoods in Taiwan have two lanes, the intersections of which are usually controlled by flashing signals. Therefore, road safety authorities should replace flashing lights with normal ones to mitigate the severity of motorcycle crash injury in university neighborhoods. Furthermore, young adults should be well educated about the right of way and the importance of defensive driving. No signal condition was found to have a marginally significant negative association with severe RTIs. As the study sample was selected from an urban environment, our results may differ from previous studies examining nationwide crashes [54]. Further research may attempt to examine the interactions between the signal status, traffic violations by motorcyclists and crash partners, vehicle maneuvers, and injury severity.

Single-motorcycle crashes can be due to loss of control, falling during a turn, or sudden motorcycle deceleration [55,56], which result in a high risk of severe injury [57]. RTIs involving collision with stationery objects are also severe [46,58]; Nunn indicated that they often involve high-speed impact and sudden deceleration [59]. A possible explanation is that traffic in university neighborhoods is frequently chaotic because of the large number of pedestrians and illegally parked motor vehicles. Motorcyclists in developing countries such as Malaysia [47], Pakistan [60], and Sri Lanka [61] face similar environments; studies from these countries have also reported single-motorcycle crashes as a risk factor for severe or fatal injury. Drunk driving, drowsy driving, and commercial transportation were also reported relating to single-motorcycle crashes [62,63,64]. Therefore, the relevant authorities should pay attention to the issue of drowsy driving, drunk driving, and food delivery driving. Strengthening road safety by removing street-side obstacles is also critical for mitigating motorcycle crash injury in university neighborhoods.

Adverse weather is generally considered a risk factor for severe motorcycle RTIs because of poor driving conditions [65]. However, in our study, adverse weather was a protective factor for severe RTIs, and some studies have also obtained similar results [44,66,67]. Pai et al. suggested that motorcyclists are driving recklessly in fine weather, which may lead to increased crash severity [67]; on the other hand, adverse weather could be a precautionary signal for riders being more cautious. Furthermore, most of the vendors in university neighborhoods close down on rainy days, so the driving environment may be safer in adverse weather conditions. On adverse weather days, people are more inclined to take mass transportation systems, causing a reduction in the number of motorcyclists, thereby potentially affecting our results.

Our results indicate that a shorter time to hospital was associated with a higher ISS. Because of the convenience and affordability of National Health Insurance, which covers almost 99% of the population in Taiwan, most patients with RTI visit the ED for medical attention [68]. The individuals with light injuries may be interviewed by the police before arriving at the ED, whereas those with more severe injuries are rapidly transported to the hospital in public ambulances, thus shortening the time to hospital [69]. RTIs in rural areas tend to have higher injury severity [12] and longer time to hospital [70]. However, we did not analyze the RTIs in rural areas.

We also noted a higher risk of severe injury among female motorcyclists, which has rarely been noted in the Malaysian study [71]. Because motorcycles are extensively used for commuting and daily activities in Taiwan, the proportion of female motorcyclists is much higher than that in other countries [72]. In 2020, the male-to-female rider ratio was close to 1.05/1 [73]. Our study revealed a ratio of approximately 1.6/1, whereas this ratio has ranged from 3.8 to 28.7 in other reports [74,75]. A Brazilian study reported that female motorcyclists were more vulnerable to RTIs [76]. Our subgroup analysis indicated an increased risk of severe injury in females aged 18 and 24 years, which is consistent with the results of the overall cohort. Further in-depth exploration is essential to investigate the association between sex and injury severity in motorcycle crashes in university neighborhoods.

Drunk driving elevates the risk of severe injury [5]. In Taiwan, random sobriety checkpoints have been used for motorists since 1996 [77]. Lowering the alcohol concentration threshold for blood screening in the ED has indicated a decline in drunk driving cases [78]. However, the sobriety checkpoints in Taiwan primarily target car drivers. Therefore, road safety authorities should implement strategies such as sobriety checkpoints for motorcyclists in university neighborhoods and screening of blood alcohol among injured motorcyclists in the nearby ED. These strategies should prioritize middle-aged and older adults because drunk driving is not common among young adults in university neighborhoods.

We compared our study results with the studies conducted in other developing countries in Southeast Asia that are geographically similar and equally highly dependent on motorcycles for commuting and transportation. Although motorcycles are the main transport tools in developing countries, the severity of motorcycle crash injuries varies depending on the economic development, urban planning, and transportation policies [72]. In terms of the time of the crash, studies in Vietnam [79], Thailand [80], and Malaysia [81] showed a higher risk of severe injury arising from motorcycle crashes that occur between night and early morning, while our study showed that early morning was a risk factor for severe injury within the whole cohort, and late-night and afternoon were significant times for drivers aged 18–24. Our study also analyzed the signal status and found that flashing signals increased the risk of a severe motorcycle crash injury, which was not observed in the studies mentioned above. In terms of the collision partners, the results of our study showed that a single-motorcycle crash was associated with the risk of a severe injury, which is consistent with the results of the study in Thailand [80]. In terms of the road alignment, the Malaysian study [81] showed that junctions were one of the risk factors that increase the severity of injuries, while no significant associations were found when comparing straight roads with curved roads or roundabouts, which is consistent with our study. Finally, although unlicensed driving was a significant risk factor in the Vietnamese and Malaysian studies [79,81], this association could only be observed from the univariate analysis in our study.

Since this study was a multicenter study including three metropolitan areas, namely, New Taipei City, Taipei City, and Tainan City, the study’s results can be extrapolated to other metropolitan areas in Taiwan but not rural ones. For other countries or areas with similar economic development and heavy motorcycle use for daily activities, the study’s results can be extrapolated to a certain extent, but cautious interpretation is necessary because of differences in healthcare systems and geographical variations. Since the proportion of Taiwanese high school graduates attending universities was almost 90% in 2017 [19], the subgroup analysis sample, namely, the 18–24-year-old adults injured in a motorcycle accident in a university neighborhood, can considerably represent university students.

We analyzed the data of 4751 motorcyclists with RTIs, making our analysis the first with a large sample size to report an association between environmental factors in university neighborhoods and injury severity among motorcyclists. As far as we know, this is the first study to investigate the association between environmental factors and motorcycle crash injury severity in university neighborhoods in Taiwan. Integrating the hospital dataset is crucial for obtaining more accurate information on motorcyclists’ injury severity. Considering our limited resources, we invited five level-I trauma centers in three metropolitan areas. It was challenging to obtain the medical data from these hospitals. By connecting the PTAD and hospital dataset, our study obtained more accurate information on the patients’ injury severity. We collected one year of motorcycle crash data verified by the National Police Agency, and the results of this study provided helpful information for the road safety authorities and researchers.

### 4.1. Limitations

Our study excluded RTAs in rural areas because the driving environment differs from urban areas. Future studies should consider the characteristics of rural RTAs. The dataset used in this study was pre-established for road safety research [18] and does not include data to identify university students. Thus, we selected the age group of 18–24 years (young adults) as a surrogate. In addition, we defined university neighborhoods as areas where university students frequently move. However, the movement of university students is not limited to such areas. Future studies should use a dataset containing information on university student status to yield more concrete results. Because of the high compliance with the compulsory helmet law in Taiwan [82], it was not feasible to analyze the effect of not using a helmet in this study. Finally, patients’ baseline data preceding their RTA events to contrast with the crash conditions were not available in the dataset. Therefore, this study only highlights risk factors for a severe crash for motorcyclists who crash.

### 4.2. Practical Implications

Driving a motorcycle in university neighborhoods during the early morning (4:00 a.m.–7:59 a.m.) is a risk factor for serious injuries. Therefore, the road safety authorities should encourage the public to use mass transportation for commuting and school, especially in the Greater Taipei metropolitan area, where a complete mass transportation system has been established. For young adults in the subgroup analysis, late at night (12:00 a.m.–3:59 a.m.) is a risk factor for serious injuries among motorcyclists. So, the relevant authorities, including the supervisors of universities, should pay attention to the issue of drowsy driving, drunk driving, food delivery driving, and road lighting during this period. For traffic signals, we recommend that road safety authorities replace flashing traffic signals with normal traffic signals in university neighborhoods to mitigate serious motorcyclist injuries. Inexperienced motorcyclists should be educated about the right of way and be more vigilant when passing the intersection with flashing signals. Finally, concerning drunk driving causing serious injuries, we recommend that law enforcement officers extend the sobriety checkpoints for roadside alcohol tests to early morning in university neighborhoods and actively test the injured patients of motorcycle accidents in the EDs near the universities.

## 5. Conclusions

- Although motorcycle crash injury is serious, especially among young adults, data on environmental factors affecting injury severity among motorcyclists in university neighborhoods are limited.
- Our study is the first to investigate the association between environmental factors and motorcycle crash injury severity in university neighborhoods in Taiwan.
- A university neighborhood is defined as an area with the most movement of university students.
- The subgroup analysis sample, namely, the 18–24-year-old adults injured in a motorcycle accident in a university neighborhood, can considerably represent university students.
- The significant risk of severe injury while driving in the early morning may reflect the high motorcyclist volume and chaotic traffic conditions during this period in university neighborhoods.
- Our data reveal actionable targets for mitigating RTIs in university neighborhoods, namely, flashing signals at intersections and roadside obstacles.
- Single-motorcycle crashes and drunk driving are significant risk factors for severe motorcycle crash injury in university neighborhoods.
- Adverse weather does not increase the risk of severe motorcycle crash injuries in university neighborhoods.
- The protective effect of longer time to hospital indicates the effectiveness of urban emergency medical services in Taiwan.
- In our study results, female motorcyclists are significantly associated with severe motorcycle crash injury in university neighborhoods, which may be due to the high proportion of female motorcyclists in Taiwan. Further in-depth research is necessary.
- The results of the subgroup analysis may reflect lifestyle habits of young adults in university neighborhoods, such as not engaging in drunk driving and frequently driving in the afternoon and late at night.

## Figures and Tables

**Table 1:** Results of univariate analysis between patients with ISS < 8 and ISS ≥ 8.

Variables	ISS < 8 (*n* = 4 238)		ISS ≥ 8 (*n* = 513)		Simple Logistic Regression	
	*n*	%	*n*	%	OR (95% CI)	*p* Value
Sex						
Men	2634	90.30	283	9.70	0.749 (0.623–0.901)	0.002
Women	1604	87.46	230	12.54	1.335 (1.110–1.605)	0.002
Age group (year)						
<24	1714	90.78	174	9.22	0.756 (0.623–0.917)	0.004
25–44	1476	91.17	143	8.83	0.723 (0.590–0.886)	0.002
45–64	842	85.22	146	14.78	1.605 (1.306–1.972)	<0.0001
≥65	206	80.47	50	19.53	2.114 (1.529–2.921)	<0.0001
Drunk driving						
No	4213	89.33	503	10.67	0.298 (0.142–0.625)	0.001
Yes	25	71.43	10	28.57	3.352 (1.601–7.019)	0.001
Cylinder capacity (cc)						
<250	4200	89.21	508	10.79	0.919 (0.360–2.346)	0.860
≥250	38	88.37	5	11.63	1.088 (0.426–2.776)	0.860
License status						
Licensed	4024	89.48	473	10.52	0.629 (0.443–0.893)	0.010
Unlicensed	214	84.25	40	15.75	1.590 (1.120–2.258)	0.010
Weather						
Fine	3001	88.53	389	11.47	1.293 (1.045–1.599)	0.018
Adverse	1 237	90.89	124	9.11	0.773 (0.625–0.957)	0.018
Day of the week						
Weekday	2955	89.03	364	10.97	1.061 (0.867–1.298)	0.567
Weekend	1 283	89.59	149	10.41	0.943 (0.771–1.153)	0.567
Time of crash						
12:00 a.m.–3:59 a.m.	181	87.44	26	12.56	1.197 (0.785–1.824)	0.404
4:00 a.m.–7:59 a.m.	378	86.10	61	13.90	1.379 (1.034–1.838)	0.029
8:00 a.m.–11:59 a.m.	1147	89.82	130	10.18	0.915 (0.741–1.129)	0.406
12:00 p.m.–3:59 p.m.	831	88.31	110	11.69	1.119 (0.894–1.400)	0.325
4:00 p.m.–7:59 p.m.	966	89.94	108	10.06	0.903 (0.722–1.130)	0.373
8:00 p.m.–11:59 p.m.	735	90.41	78	9.59	0.855 (0.663–1.101)	0.225
Rush hour						
Yes (7:00 a.m.–9:59 a.m.; 6:00 p.m.–8:59 p.m.)	1564	90.20	170	9.80	0.847 (0.698–1.029)	0.095
No (10:00 a.m.–5:59 p.m.; 9:00 p.m.–6:59 a.m.)	2674	88.63	343	11.37	1.180 (0.972–1.433)	0.095
Speed limit (km/h)						
≤50	4175	89.23	504	10.77	0.845 (0.418–1.709)	0.639
>50	63	87.50	9	12.50	1.183 (0.585–2.394)	0.639
Road alignment						
Straight road	1712	89.82	194	10.18	0.897 (0.743–1.084)	0.260
Curved road	86	86.00	14	14.00	1.355 (0.765–2.402)	0.298
Crossroad/Roundabout	2440	88.89	305	11.11	1.081 (0.897–1.302)	0.416
Road surface						
Dry	3480	88.89	435	11.11	1.215 (0.943–1.565)	0.133
Wet/Slippery	758	90.67	78	9.33	0.823 (0.639–1.061)	0.133
Sight						
Good	4176	89.21	505	10.79	0.937 (0.446–1.968)	0.864
Bad	62	88.57	8	11.43	1.067 (0.508–2.241)	0.864
Signal status						
Normal	1707	89.00	211	11.00	1.036 (0.860–1.248)	0.710
Flashing	147	79.89	37	20.11	2.163 (1.490–3.141)	<0.0001
No	2384	90.00	265	10.00	0.831 (0.692–0.998)	0.048
Collision partner						
Pedestrian	137	91.33	13	8.67	0.778 (0.437–1.385)	0.394
Vehicle	3855	89.48	453	10.52	0.750 (0.562–1.001)	0.051
None	246	83.96	47	16.04	1.637 (1.181–2.268)	0.003
Road width (m) median (IQR)	10	(11)	11	(11)	0.994 (0.984–1.005)	0.281
Time to hospital (min) median (IQR)	33	(38)	28	(14)	0.984 (0.972–0.995)	0.005

CI, confidence interval; IQR, interquartile range; ISS, injury severity score; OR, odds ratio.

**Table 2:** Results of a multivariate analysis between independent variables and severe injury (ISS ≥ 8).

Variables	*β*	SE	OR	95% CI	*p* Value
Sex					
Men	Reference				
Women	0.274	0.097	1.315	1.087–1.592	0.005
Age group (year)					
<24	0.083	0.121	1.086	0.857–1.376	0.493
25–44	Reference				
45–64	0.560	0.127	1.751	1.365–2.247	<0.001
≥65	0.935	0.184	2.547	1.776–3.653	<0.001
Drunk driving					
No	Reference				
Yes	1.071	0.391	2.918	1.355–6.281	0.006
Weather					
Fine	Reference				
Adverse	−0.250	0.111	0.779	0.626–0.969	0.025
Time of crash					
12:00 a.m.–3:59 a.m.	0.359	0.238	1.432	0.899–2.282	0.131
4:00 a.m.–7:59 a.m.	0.342	0.170	1.408	1.010–1.963	0.043
8:00 a.m.–11:59 a.m.	Reference				
12:00 p.m.–3:59 p.m.	0.182	0.139	1.200	0.913–1.577	0.191
4:00 p.m.–7:59 p.m.	0.028	0.140	1.028	0.782–1.353	0.841
8:00 p.m.–11:59 p.m.	0.047	0.156	1.048	0.772–1.421	0.765
Signal status					
Normal	Reference				
Flashing	0.677	0.203	1.968	1.324–2.927	0.001
None	−0.166	0.100	0.847	0.697–1.030	0.096
Collision partner					
Pedestrian	Reference				
Vehicle	0.217	0.299	1.242	0.691–2.232	0.469
None	0.765	0.336	2.150	1.112–4.155	0.023
Time to hospital (per 10 min)	−0.001	0.001	0.987	0.976–0.998	0.018

Hosmer & Lemeshow test: *p* = 0.459. CI, confidence interval; ISS, injury severity score; OR, odds ratio; SE, standard error.

**Table 3:** Subgroup analysis between independent variables and severe injury (ISS ≥ 8) among young adults.

Variables	*β*	SE	OR	95% CI	*p* Value
Sex					
Men	Reference				
Women	0.441	0.167	1.555	1.121–2.157	0.008
Time of crash					
12:00 a.m.–3:59 a.m.	0.749	0.335	2.116	1.096–4.083	0.026
4:00 a.m.–7:59 a.m.	0.586	0.364	1.797	0.881–3.667	0.107
8:00 a.m.–11:59 a.m.	Reference				
12:00 p.m.–3:59 p.m.	0.559	0.244	1.749	1.085–2.819	0.022
4:00 p.m.–7:59 p.m.	0.180	0.266	1.198	0.711–2.016	0.497
8:00 p.m.–11:59 p.m.	0.210	0.264	1.233	0.735–2.071	0.427
Signal status					
Normal	Reference				
Flashing	1.069	0.297	2.913	1.628–5.213	<0.001
None	−0.047	0.174	0.954	0.679–1.342	0.788
Time to hospital (per 10 min)	−0.002	0.001	0.976	0.954–0.999	0.038

Only significant variables (*p* < 0.05) are shown in Table 3. Hosmer and Lemeshow test: *p* = 0.121. CI, confidence interval; ISS, injury severity score; OR, odds ratio; SE, standard error.

## Data Availability

The data that support the findings of this study are available from the corresponding author upon reasonable request.
